# Building Collective Power to Advance Maternal and Child Health Equity: Lessons from the New Orleans Maternal and Child Health Coalition

**DOI:** 10.1007/s10995-024-04000-7

**Published:** 2024-09-28

**Authors:** Iman Johnson, Dovile Vilda, Emma Allen, Desiree Boisson, Clare Daniel, Latona Giwa, Melissa Goldin Evans, Hali Ledet, Lisa Richardson, Maeve Wallace

**Affiliations:** 1 Department of Social, Behavioral, and Population Sciences, Mary Amelia Center for Women’s Health Equity Research, Tulane University School of Public Health and Tropical Medicine, New Orleans, LA USA; 2 Newcomb Institute, Tulane University, New Orleans, LA USA; 3 St. Georges University, St. George, Grenada; 4 New Orleans Breastfeeding Center, Birthmark Doula Collective, New Orleans, LA USA; 5 Vanderbilt University School of Nursing, Nashville, TN USA; 6 Institute of Women & Ethnic Studies, UNO Research and Technology Foundation, Inc., New Orleans, LA USA; 7 Department of Social, Behavioral, and Population Sciences, Mary Amelia Center for Women’s Health Equity Research, Tulane University School of Public Health and Tropical Medicine, 1440 Canal St. | Suite 2210, New Orleans, LA 70112 USA

**Keywords:** Maternal child health, Coalition, Health equity, Collective power, Reproductive justice

## Abstract

**Objectives:**

The New Orleans Maternal Child Health Coalition convenes to support and amplify the work of New Orleans-based individuals and organizations working to reduce disparities and protect the health of birthing families in the New Orleans area. The objectives of this qualitative study were to identify successes, challenges, and areas of growth for the Coalition and develop broadly generalizable recommendations for similar groups seeking to mobilize and advance health equity in their own communities.

**Methods:**

Using purposive sampling, we conducted semi-structured interviews with 12 key informants from within and outside of the Coalition. Interviews were transcribed verbatim, and data was analyzed using inductive and deductive coding approaches.

**Results:**

We identified themes relating to the barriers and facilitators to the maintenance of the Coalition, as well as opportunities to advance the mission of the Coalition. Some themes included structural- and systemic-level barriers to achieving the mission, varying perspectives on the effectiveness of the Coalition, opportunities to enhance the operations of the Coalition’s work, and opportunities to involve other individuals, particularly those with lived experience, and non-MCH related sectors in Coalition’s work.

**Conclusions for Practice:**

As the maternal health crisis continues, coalitions like the New Orleans MCH Coalition provide a vehicle to amplify the mission-driven work of people and organizations. Recommendations put forth by the Coalition can also be utilized by coalitions in other jurisdictions.

**Supplementary Information:**

The online version contains supplementary material available at 10.1007/s10995-024-04000-7.

## Significance

What is Already Known on this Subject?

What Does this Study Add?

Black–White disparities in maternal and child health (MCH) have a longstanding history within the United States (US) and these disparities are mirrored in New Orleans, LA. The New Orleans Maternal and Child Health Coalition was founded to support the collective efficacy of numerous individuals and organizations to protect the health of birthing families in the community.

We identified the key barriers and facilitators to achieving the Coalition’s mission and building collective power to achieve MCH equity in the greater New Orleans area. Such evidence is a crucial contribution during this time when rates of maternal mortality and morbidity continue to rise throughout the country. This study amplifies the importance of community-driven work to address disparities in MCH and enhance health equity.

**Supplementary Information:**

The online version contains supplementary material available at 10.1007/s10995-024-04000-7.

## Introduction

The US has one of the highest maternal mortality rates in the industrialized world (Kassebaum et al., 2014). The burden of these losses is experienced three to four times more frequently among Black families compared to non-Hispanic (NH) White families (Centers for Disease Control and Prevention, 2022a). Similar trends are seen in infant mortality as Black infants are twice as likely to die during their first year than NH White infants (Centers for Disease Control and Prevention, 2022b; Rossen et al., 2016). National trends in maternal and child health (MCH) are mirrored or worsened in the City of New Orleans where preterm birth (PTB), low birth weight (LBW), and infant mortality consistently occur at rates two to three times more frequently among Black pregnant people compared to NH White people (Louisiana Department of Health, 2022). In New Orleans, similar to other American cities, the enduring legacy of structural racism—policies, practices, norms and values that privilege whiteness—underlies entrenched racial inequities in maternal and child health. Structural racism is evident in the unequal distribution of, and access to, income, education, and a broad range of health-promoting resources and opportunities, neighborhood conditions, and the generational accumulation of wealth (Broussard et al., 2018).

Originating in 2018, the New Orleans Maternal and Child Health Coalition (the Coalition hereafter) formalized a convening mechanism to support the collective efficacy of numerous individuals and organizations working to protect the health of birthing families in New Orleans. Coalitions have been key to advancing feminist and social justice causes and the health of marginalized communities for decades (Anderson et al., 2015; Bibeau et al., 1996; Khare et al., 2015; Luna, 2010; Staggenborg, 1986; Taylor, 2018). Feminist coalitions often coalesce around an opportunity or a threat (Staggenborg, 1986) and seek to foreground the needs and voices of the people most affected by an issue (Taylor, 2018). Further, health coalitions and advocacy groups have served as a mechanism for improving health outcomes within a community at different levels of the social ecological model (Butterfoss, 2007; Butterfoss et al., 1993). Coalitions bring diverse stakeholders together around various issues for which they can collectively strategize, share resources, and execute health interventions that single agencies or individuals would be unable to do on their own. The Coalition embodies these characteristics. Founded on both the threat of the maternal mortality crisis in Louisiana and the opportunity that increased media attention to this issue presented (Villarosa, 2018), the Coalition seeks to prioritize the perspectives of Black birthing people and those who provide them care.

The Coalition is guided by the principles of the Black feminist framework of reproductive justice (Ross & Solinger, 2017) which is reflected in its mission “to improve outcomes, experiences, and access to quality, respectful care during pregnancy, birth, and the postpartum period by centering the experiences of Black birthing people and their infants in New Orleans” (New Orleans Maternal & Child Health Coalition, 2023). The membership consists of over 200 individuals representing academia, city and state government, community-based organizations, healthcare providers, and birthing people. The Coalition is convened through a major academic institution and a non-profit in the New Orleans area. The Coalition also maintains an advisory board. Prior to 2023, the Coalition operated without any dedicated funding. Since then, it was awarded a grant from a foundation to hire a fulltime coordinator. Since its founding, the Coalition has advocated for changes in policy and practice at the city and state level and mobilized the advocacy based through organizing efforts and the use of digital and traditional media.

Given the pressing nature of this work and the need to assess its effectiveness, the Coalition assembled a core team of Coalition members—including representatives from both academic and community partners—to turn a critical eye on ourselves and gather insights from key informants within and outside of the Coalition. Drawing on qualitative interviews with key stakeholders, the aims of this qualitative study were twofold: (1) to identify successes, challenges, and areas of growth for the Coalition, and (2) to develop broadly generalizable recommendations for this Coalition and similar groups seeking to mobilize and advance health equity in their own communities.

## Methods

### Selection of Key Informants

Purposive sampling was used to select a group of key informants. Initially, members of the research team established a list of 24 Coalition members and Coalition adjacent MCH professionals as potential key informants. This list prioritized key informants that reflected a range of experiences within the greater New Orleans MCH landscape, various degrees of involvement with the Coalition, and a diversity of professional perspectives representing the non-profit sector, medical and nursing professionals, academics, and community members with lived experiences. Potential key informants were contacted via email with an invitation to participate. Out of our initial goal to recruit 10–15 key informants, 12 of those invited agreed to be interviewed.

### Data Collection

Semi-structured interviews were the primary method of data collection. The research team developed an interview guide (see Supplement A) to address five domains important to this inquiry: (1) participant background and sociodemographic characteristics, (2) perspectives on MCH inequities, (3) Coalition history and involvement, (4) Coalition’s mission and work, and (5) internal processes and procedures. All interviews were conducted via Zoom or phone from September through November 2021 by a trained interviewer on the research team. All interviews were audio-recorded with participants' consent and typically lasted between 30 and 90 min. Participants received an honorarium for their involvement as a token of appreciation for their time.

### Analysis

All interviews were transcribed verbatim, checked for accuracy, and anonymized. Data analysis was initiated early during the data collection process with brief case summaries prepared for each key informant as interviews were transcribed. These summaries were designed to synthesize key sociodemographic data related to key participants’ involvement in the MCH field and the Coalition. Interview data was analyzed and interpreted by two experienced members of the research team using deductive (i.e., an interview guide was used to establish a primary coding framework) and inductive (i.e., coders continued to add new themes and sub-themes as they emerged in the data) approaches to coding for thematic analysis (Fereday & Muir-Cochrane, 2006). The coders met on a weekly basis to compare and discuss the analysis of interview data, and the development of individual codebooks. Based on these discussions, the coders reconciled their codebooks and collapsed smaller codes into larger thematic and descriptive codes. Saturation of themes (the point at which no new themes were identified in the transcripts) was achieved, and a final coding scheme—comprised of 28 key conceptual and descriptive categories—was subsequently developed to facilitate the labelling, sorting and synthesis of data in NVivo 12 plus software. After analysis of all 12 interviews, findings were brought to the larger research team for further discussion on themes that might have been overlooked during initial and subsequent rounds of coding. Each key informant was assigned a pseudonym, and all potentially identifying information (names of people, places and other details) were removed from the narrative excerpts presented in this paper.

## Results

The final sample included 12 key informants aged 29 to 69 years old. All key informants identified as female; 9 of the 12 identified as African American or NH Black, and the remaining informants identified as NH White. Most participants reported working in the non-profit sector and/or as birth workers, two informants held academic positions, and one was working at a governmental institution (Table 1).

**Table 1:** Sociodemographic characteristics of the key informants (N = 12)

Interview #	Age	Gender	Type of work	Race and ethnicity	Joined MCH Coalition	Duration of residency in New Orleans, LA	Coalition role
Interview 1	Under 40	Female	Non-Profit/Lived Experience	African American or NH Black	2018	Does not live in New Orleans, LA	General Coalition member
Interview 2	Over 40	Female	Government	NH White	Unknown	> 10 years	General Coalition member
Interview 3	40 Plus	Female	Non-Profit	African American or NH Black	2018	> 30 years	General Coalition member
Interview 4	Under 40	Female	Non-Profit	African American or NH Black	2019	Since 2018	Advisory Board member
Interview 5	Over 40	Female	Birthworker/Advocacy	NH White	2020	> 30 years	General Coalition member
Interview 6	Over 40	Female	Academia	NH White	2018	> 10 years	General Coalition member
Interview 7	Over 40	Female	Academia	African American or NH Black	2018–2019	Born in New Orleans, LA	General Coalition member
Interview 8	Over 40	Female	Non-Profit	African American or NH Black	2018	> 20 years	Advisory Board member
Interview 9	Over 40	Female	Non-Profit/Birthworker	African American or NH Black	Unknown	Unknown	General Coalition member
Interview 10	Under 40	Female	Birthworker	African American or NH Black	2018	> 10 years	General Coalition member
Interview 11	Under 40	Female	Birthworker	African American or NH Black, Native American	2018–2019	Since 2010	General Coalition member
Interview 12	Over 40	Female	Birthworker	African American or NH Black	Unknown	Since 2001	General Coalition member

*LA* Louisiana, *NH* non-Hispanic

Table 2 outlines key themes and sub-themes pertaining to the study aims. The following sections detail specific points raised under these themes and sub-themes and conclude with recommendations that emerged from across the interviews.

**Table 2:** Study aims, themes and sub-themes

	Study aim	Key theme(s)	Sub-themes
1	To document the development, processes, challenges and opportunities that emerged from the successful establishment of the Coalition	Creation and maintenance of the Coalition: facilitators and barriers	**•** Perceived role and the effectiveness of the mission of the Coalition **•** Benefits of being involved in the Coalition **•** The main challenges of achieving the mission
2	To identify areas of growth for the Coalition as well as make recommendations for its work moving forward	Areas of growth and future directions	**•** Areas of improvement **•** Opportunities to advance Maternal and Child Health (MCH) equity **•** Expanding the scope of the Coalition’s work

### Creation and Maintenance of the Coalition: Facilitators and Barriers

Five key informants reported having joined the Coalition during its inception in 2018, whereas the remaining participants became involved in the Coalition in the following years through their mutual connections, joint advocacy activities, or other MCH-related work. Most key informants identified themselves as “*not very active*” or “*inactive*” members and talked about the changing nature of their involvement in the Coalition’s work (“*I was definitely much more involved at the beginning of the founding*”, Interviewee #6)*.* However, all of them felt they had a good grasp of the ongoing work of the Coalition through weekly newsletters and attendance at regular meetings and calls. Several participants identified different ways in which the Coalition was seen as maintaining a “*steady growth*” (Interviewee #1) pattern and improving its activities over years.

Key informants cited relationships within the Coalition as a facilitator to doing MCH-related work. Most key informants were extremely positive when discussing the nature of relationships with other partners and member organizations, describing their connections as “*a sisterly-like bond*” (Interviewee #1), having “*each other’s back when it comes to moving initiatives*” (Interviewee #3), and utilizing a collective power/voice to reach common goals. A few participants described their relationship with other Coalition members as ranging from very strong to familiar depending on their work outside of or prior to the Coalition, whereas one participant described MCH work in New Orleans generally as “*territorial*” (Interviewee #11).

#### Perceived Role and Effectiveness of the Coalition’s Mission

Many key informants emphasized the importance and impact of collective work, advocacy efforts, and resources in advancing the mission of the Coalition. The professional diversity within the Coalition was frequently perceived as the major organizational strength and facilitator in coordinating efforts across the broader MCH network (“*We have so many people, like there are policy people we have researchers we have you know birthing people we have birth workers, like, OBs or you know doulas or midwives who are represented in the Coalition, we have representatives from health departments both the local and state health departments and then other organizations doing the work so I think that’s a strength*”, Interviewee # 4).

Other informants expressed more critical or ambiguous views on the effectiveness of the Coalition’s work or abstained from commenting, referring to themselves as not qualified or knowledgeable enough to evaluate. One key informant commented on the Coalition as being reactive in its pursuit of the mission rather than more proactive in its advocacy actions.

#### Benefits of Being Involved in the Coalition

Most informants reported utilizing collective voice, accessing or sharing community resources, and expanding collaborations and professional networks as the key personal and organizational benefits of being involved in the Coalition. These aspects were seen as especially critical in contributing to policy and advocacy efforts (“*[I]t is really cool to have a common agenda, we can have a ****collective voice****, I do like that idea of it as well as having a common voice and you know we put out a white paper or you know things like that, there are 60 organizations standing behind this you know*”, Interviewee #10) (for a list of recent successful legislative advocacy efforts, see Table 3).

**Table 3:** Select successful MCH Coalition-supported legislative efforts (2022–2023)

Bill number	Bill summary	When passed
SB 135	Medicaid Reimbursement for licensed and certified midwives	2023
HB 272	Insurance coverage for doulas	2023
HB 516	Requires each governing authority of a public high school to adopt policies regarding attendance, breastfeeding, and child care for students who are pregnant or parenting	2022
HB 651	Medicaid and Insurance coverage for prescribed human milk	2022
HB 90	The creation of the Office of Women’s Health within the Department of Health	2022
HB 516	Provides universal perinatal mood disorder screenings with primary care doctors	2022

The Coalition was also perceived as a useful resource hub serving its members different functions, including providing information about local services and events, providing practical support, sharing expertise and resources (“*[T]hey’ve helped me actually write a couple grants and they’ve helped my efforts as far as like getting supplies for families* <*…*> *at the beginning of the [COVID-19] pandemic*”, Interviewee #10), and orienting people towards policy efforts. Furthermore, connecting with other Coalition members, creating partnerships and networking were identified by most participants benefits of being involved in MCH Coalition.

Among other benefits, the key informants mentioned accessing different perspectives (especially patient/birthing people), being able to better understand MCH issues, and observing Black women’s leadership.

#### The Main Challenges of Achieving the Mission

Participants identified numerous challenges of advancing the mission of the Coalition. Most of them were linked to either the broader system-level barriers encountered in improving MCH equity in New Orleans and Louisiana (i.e., systemic racism, systemic bias, multiple intersecting social determinants of health) or mission-related and operational issues faced by the Coalition in its work. Ensuring racial/ethnic and professional diversity within the Coalition and centering on the common goal (i.e., improving health and lives of birthing people) were seen as strategies already embedded in Coalition’s structure and work.

One of the challenges identified was the need to redefine or clarify the mission, and to underscore advocacy efforts as a specific strategy for advancing maternal and child health equity: “<*…*> *advocacy, especially policy-based advocacy, is incredibly time intensive and labor intensive* <*…*>*, right? To build those relationships, develop them, pick the right people to help move things forward, all of that becomes really challenging and so I think [it is important] to have whether it be a subgroup or advocacy wing*” (Interviewee #2).

Other operation-related challenges included addressing redundancies in activities across partnering organizations and among the Coalition members. Additionally, many informants recognized the limited capacity/commitment and time investments among the Coalition members (“*coalitions are not easy to maintain, it’s work*”, Interviewee #9). Proposed solutions included facilitating participation through virtual meetings, and incentivizing people for their work in the Coalition. One informant highlighted the need to have a “*centralized person*” (Interviewee #10) who would represent the Coalition in advocacy and policy work.

### Areas of Growth and Future Directions

#### Areas of Improvement for the Coalition

Key informants highlighted two areas as critical in improving the Coalition’s operations, namely improving member engagement and becoming more task-oriented. Most key informants recognized the varying engagement among Coalition members as “*a challenge*” which was attributed to a general pattern of engagement observed across other Coalitions and organizations of a similar nature (“*I think every organization struggles with engagement of their members,* <*…*> *you want to communicate enough to keep folks engaged and informed, but not so much that they become overwhelmed and ignore what you do*”, Interviewee #6). Low or fluctuating engagement was attributed to the nature of the Coalition as a volunteer-based organization in which most members have full-time commitment to MCH-related work and thus do not have the capacity to be fully engaged. As a result, participants recognized the extra effort that the Coalition’s leadership was putting in to keep the Coalition moving forward.

Several solutions were proposed to keep the members more engaged, including strengthening cross-sector relationships and providing opportunities for more engagement in between Coalition meetings, incentivizing Coalition members, avoiding overlapping activities across other organizations, and maintaining flexibility of meetings (i.e., having meetings on a video conferencing platform, considering different meeting hours for members with conflicting commitments). One participant proposed to increase member participation by having them take ownership of specific tasks and “*dividing labor*” (Interviewee #2) within the group.

Revising the structure of the Coalition and becoming more task-oriented across other activities was also seen as a way of addressing operational challenges. Several key informants proposed to “*map out the expertise within the Coalition*” (Interviewee #7) by compiling a resource database detailing each members area of expertise and potential contribution to the Coalition. Additionally, two key informants talked about the different domains within the Coalition (i.e., policy advocacy, research, fundraising and grant writing) and suggested forming different work groups to address these needs*.*

#### Opportunities to Advance MCH Equity

Key informants identified numerous opportunities for the Coalition to advance MCH equity in New Orleans and beyond, including (1) to increase representation from other groups and sectors, (2) enhance utilizing available institutional, human, and other resources, (3) identify new avenues to disseminate information from and market the Coalition, and (4) increase engagement with, and education of, other sectors and people on the outside.

Most participants emphasized elevating marginalized voices and including different perspectives at the table as one of the key strategies to improve the Coalition’s effectiveness in enhancing MCH health equity. Almost all respondents spoke about the need to reflect who is represented by the Coalition and who is missing among the members (“*Is there a way for the Coalition to think creatively or differently about who’s in the room?*”; “*Can we commit ourselves fully to this idea of elevating marginalized voices*?”, Interviewee #2). Several key populations were identified as potentially missing from these conversations and under-represented in the Coalition, including birthing people with lived experiences (Black and Brown, low-resource, and otherwise marginalized birthing people) and their partners/family members, people from the LGBTQ+ community, and different service providers*.* Including these voices was seen to as a critical attempt to evaluate community needs and provide a feedback mechanism.

The idea of intentionality was reiterated when reflecting on the need to enhance engagement with other sectors, including those outside of public health. Several participants talked about the potential ways in which the Coalition might increase its activities in engaging with those “*on the outside*” (Interviewee #4), instead of uniting people doing MCH-related work—which was seen as “*preaching to the choir*” (Interviewee #8). Intentional and stronger engagement with the medical professionals, policy makers/legislators and the community were seen as particularly critical in forging more cross-sector collaborations. However, as one participant noted, bringing other sectors to the table may raise some internal questions and will require redefining the Coalition’s key strategies and objectives accordingly (“*[H]ow does this feed into their goals and initiatives or value add?*”, Interviewee #8).

Another way to advance MCH health equity work was identifying new avenues to disseminate information and potentially bring new people into the Coalition. Participants mentioned using social media and noting its effectiveness. One participant noted that the Coalition has an opportunity to change the narrative around maternal health: “*We only hear about the bad stuff, but let’s put out some positive media, you know, stories that are good, we’re doing something right or good, let’s publicize that, let’s get it out there*” (Interview #9)*.*

Finally, a more strategic use of already available resources (including institutional affiliation, collective expertise, and successful programming examples from other states and cities) was often identified as a way of enhancing the Coalition’s role in MCH work. The Coalition’s affiliation with the private research University was seen as a prominent resource that is under-utilized in the Coalition’s work (e.g., “*I would be very open to considering how do we draw more, [institution] MPH students into that potentially?*”, Interviewee #7). Seeing the Coalition as an asset, particularly in educating and involving more students, participants emphasized the networking prospects and an opportunity to observe Black leadership (“*I’m so grateful to be able to work with these powerhouses and for our students to be able to learn from them*”, Interviewee #6). However, affiliation with a private predominantly white university was seen as a privilege shared only by its students, and some key informants emphasized the need to increase engagement with students from Historically Black Colleges and Universities (HBCUs) both in New Orleans and around the state.

#### Expanding the Scope of the Coalition’s Work

When reflecting on the question of whether, and to what extent, the Coalition should expand its work geographically to other areas of Louisiana, the majority of participants noted the critical need for greater efforts throughout the state, particularly in relation to state-level legislative and advocacy work. However, key informants were split on the role of the Coalition in expanding and facilitating the required work in other locations, ranging from those advocating for “*should definitely expand*” (Interviewee #9), strategically partner or act as a pilot for other jurisdictions, to questioning if the currently Coalition has the capacity to expand.

Participants who believed the Coalition should expand its geographic reach noted the MCH needs of other cities and locations in Louisiana, while also recognizing that New Orleans is substantially different from other parts of the state (“*I would love to see it more statewide for sure, even involve members from other regions of the state, because New Orleans is kind of isolated, you know? I mean, politically in our state*”, Interviewee #5). Other participants noted that the Coalition currently does not have the capacity to expand its work arguing that advocacy efforts take time and resources and would be difficult. However, these members saw the Coalition as an example or pilot for other locations and initiatives across the state also providing support through collaborative efforts and partnerships.

## Discussion

Racial inequities in MCH remain vast and entrenched despite decades of public health research and programming. Innovative approaches to advocacy for broad, policy-level intervention that address inequitable social conditions as root causes of poor health are needed. Coalitions such as the New Orleans Maternal and Child Health Coalition may represent a promising model for convening and building collective power and resources towards the advancement of population health equity. Literature has shown that coalitions can be a critical tool in building political power within communities (Khare et al., 2015; Wolff, 2001) and a way to influence the political process (Holm & Shaheen, 1995). Further, health coalitions not only support health promotion within communities they represent, but also advance equity and eliminate health disparities (Butterfoss et al., 1993; John et al., 2021; Reid et al., 2019).

Our analysis identified successes, challenges, and opportunities encountered in the pursuit of the Coalition’s mission. The strength of relationships—both individual and organizational—within the Coalition was identified as a central feature of the Coalition’s success to date. Strong relationships are crucial to rallying members around advocacy issues, building a collective voice, and advancing legislative work. Previous literature has cited member participation, member satisfaction, and member agency collaborations as keys to coalition success (Knight, 2015). Additionally, coalition members’ extensive expertise can be and has been leveraged to move the mission forward and address various MCH issues. Many key informants cited the Coalition’s primary communication channels—the Coalition newsletter, a weekly email informing the Coalition members about events in the community, and the action emails—as important tools for building collective power. The action emails give both active members and those who are peripherally involved the opportunity to mobilize legislation campaigns and efforts related to MCH. In addition, newsletter dissemination provides opportunities for members to share upcoming events with other members, serving as a mechanism to continuously foster internal relationships.

Key informants identified structural racism and other systemic barriers as the most salient challenges affecting the social and economic conditions that perpetuate inequities in maternal and child health. These findings echo the literature on the reasons for maternal health inequities (Brown et al., 2021; Crear-Perry et al., 2021; Taylor, 2020). These barriers also highlight ongoing calls to elevate the voices of Black pregnant and birthing people. Centering the stories of Black people is imperative to advancing health equity at large, maternal health equity, and reproductive justice (Barlow & Johnson, 2021; Blackstock & Blackstock, 2021; Houston & Walker, 2022). This approach is embedded in the mission of the Coalition, as reflected in the mission statement itself, the Coalition’s core values, and the make-up of the advisory board (the majority of advisory board members are Black women, often representing Black-led organizations that directly serve or advocate on behalf of Black birthing people) (New Orleans Maternal & Child Health Coalition, 2023). Many of the coalition members have lived experience as birthing people themselves, whether they also engage in this work as their profession or not.

Another challenge identified in this study related to varying perceptions of the Coalition’s mission, highlighting an internal tension that the Coalition will need to address in the future. Findings suggest that key informants’ ideas of whether the Coalition was effective at achieving its mission hinged on personal involvement in the Coalition’s work, and whether the key informant worked in direct services or a different area of the MCH workforce. Lesser involved key informants perceived the Coalition as less successful and made suggestions that the Coalition has already put in place or attempted (i.e., developing a membership directory).

Our findings revealed several opportunities for the Coalition to continue advancing its mission including ways to maximize Coalition capacity, leveraging affiliations with academic partners, bringing new and necessary voices to the table such as those with relevant lived experiences, and developing partnerships with non-MCH sectors that could add valuable insight to MCH issues. Mapping out key skills, experiences and coalition resources can help the Coalition move forward on previously identified priorities and maximize coalition capacity. One approach is to better update and maintain the membership directory which would include not only organizational affiliations, but also specific skills that members already have and want to build and/or expand. This would allow the Coalition’s leadership to effectively “tap” members for specific projects utilizing current skills and helping members build new ones, as well as increasing member engagement. This also encourages future collaborations amongst Coalition members and their organizations. Leveraging affiliations with research institutions and academic partners is another key strategy to moving the mission forward. Despite concerns that the affiliation with a large, primarily white, private university might prevent authentic community engagement, most key informants saw the Coalition’s affiliation with a large research university as beneficial. Leveraging the research expertise within academic partnerships can help the Coalition remain proactive by providing scientific evidence for policy advocacy as well as bringing opportunities to engage different student groups from other universities within the area (including HBCUs), allowing the Coalition to disseminate ideas to new audiences. Finally, developing partnerships with entities outside of the traditional MCH space and those with lived experience will help the Coalition to break down siloes and advance parallel progressive issues that may not directly be related to MCH, but affect social, economic, and material conditions that impact MCH populations (Roussos & Fawcett, 2000; Campbell et al., 2015).

### Key Recommendations

Information from the key informant interviews were used to develop recommendations for improving the Coalition’s internal and external operations to further build collective power amongst MCH professionals and organizations in New Orleans. These recommendations are important to the New Orleans MCH Coalition but could also be relevant for other coalitions focused on health equity and social justice. Recommendations can be seen in Table 4.

**Table 4:** Key recommendations

Internally Focused Recommendations	**•** Have a clearly defined mission that addresses the types of work the Coalition engages in **•** Develop specific strategies, processes, and procedures that will proactively address MCH equity issues within the greater New Orleans area (i.e., research activities that outline current MCH trends the Coalition could advocate for) **•** Consider the distribution of work and how the Coalition activities will be addressed and implemented **•** Maintain and enhance the Coalition’s directory that identifies group members’ expertise and their capacity **•** Leverage institutional resources to advance Coalition’s mission and associated activities (i.e., institutional or organizational funding mechanisms, student volunteers, etc.)
Externally Focused Recommendations	**•** Identify ways to connect with and incorporate non-traditional MCH sectors as well as members with lived experience, whether or not they are professionally involved in MCH-related work **•** Develop dissemination strategies beyond the current membership and identify avenues to market the Coalition more broadly and attract new membership

## Strengths and Limitations

This paper has several strengths and limitations. These findings are based on analysis of a single coalition based in a unique context and key findings may therefore have limited generalizability. However, they offer insights and recommendations that future coalitions working to improve maternal health equity may find relevant. Key informants involved in this research were selected to represent broad membership of the Coalition. Key informants have years of professional and lived experiences, as well as a deep expertise on maternal and child health issues, making them highly qualified to speak on ways to build collective power and advance maternal health equity. With that, there may be perspectives that were not captured during the interview process. Many of the members interviewed were less active in the Coalition’s work at the time of the interview and they may have been less aware of current activities and operations of the Coalition. This may have impacted the relevance of their opinion on how to address ongoing challenges or their ability to identify areas of needed growth. More active Coalition members may or may not have had more critical perspectives of the Coalition’s current work. Additionally, the COVID-19 pandemic has shifted and shaped how some members interact with the Coalition. The current virtual format may distance some members from the work, which may have influenced who decided to participate in the project.

## Conclusion

Coordinated collaborative efforts to advance MCH equity and justice are critically needed now more than ever, in light of the current and continued threats to reproductive justice (Ellis & Hicken, 2022; Wittenberg, 2022), both in Louisiana and nationwide. Coalitions such as the New Orleans MCH Coalition may be one effective strategy for replication across other jurisdictions seeking to protect and promote population health.

## Supplementary Information

Below is the link to the electronic supplementary material.

Supplementary file1 (DOCX 89 KB)

## Data Availability

The dataset generated and/or analyzed during the current study is available from the corresponding author on reasonable request. Not applicable.
