# Supplementary material for: Building Collective Power to Advance Maternal and Child Health Equity: Lessons from the New Orleans Maternal and Child Health Coalition
Source: Matern Child Health J. 2024 Sep 28;28(12):2126–36. doi: 10.1007/s10995-024-04000-7 (PMC11564310; doi:10.1007/s10995-024-04000-7)
Supplement: Supplementary file 1 — Supplementary file1 (DOCX 89 KB) [file 10995_2024_4000_MOESM1_ESM.docx]

**SUPPLEMENT A.**

**Building Collective Power to Advance Maternal and Child Health Equity**

New Orleans Maternal & Child Health Coalition Interview Field Guide

***1. Participant sociodemographic characteristics***

1. Please state your name, current affiliation and title.

a. How long have you worked in this position or capacity?

b. Have you held other MCH positions previously?

2. Where are you from?

3. If from elsewhere, how long have you lived in New Orleans?

4. If you are willing, would you mind sharing your age (approximate is fine), your racial/ethnic and gender identities?

***2. Perspectives on maternal and child health***

5. In what ways is the work that you do related to maternal and child health?

a. Is the scope of your work confined to New Orleans, or broader?

6. What do you understand to be the root or most fundamental causes of racial inequities in maternal and child health in New Orleans?

7. Generally speaking, what would you describe as the major ongoing barriers or obstacles to advancing racial equity in maternal and child health in the city?

8. How would you describe the political context for agencies, organizations, persons and coalitions engaging in an agenda to advance maternal and child health equity in New Orleans?

a. What is the level of effort, interest, willingness, collaboration, competition, silo, redundancy?

***3. Coalition history and involvement***

9. To what degree, if any, have you been involved with the New Orleans Maternal and Child Health Coalition since its founding in 2017?

a. How did you hear about the coalition? What drew you to become involved?

b. Has the nature of your involvement changed overtime?

c. How has being a member of the coalition benefitted you or your organization?

10. What is your impression of how the coalition has developed over time?

11. How would you characterize the nature of relationships or partnerships that you and/or your agency have with other members/agencies in the coalition?

***4. Coalition mission and work***

12. The mission of the New Orleans Maternal and Child Health Coalition is to “improve outcomes, experiences, and access to quality, respectful care during pregnancy, birth, and the postpartum period by centering the experiences of Black birthing people and their infants in New Orleans”

a. Do you feel the coalition has been effective at advancing its mission? Why or why not?

b. In what way do you feel the coalition does or can play a role in improving maternal and child health in New Orleans?

c. What do you see as the main challenges facing the coalition in achieving its mission?

d. What do you see as the main opportunities, or ways in which the coalition can play a role in advancing maternal and child health equity?

c. What are your thoughts on whether/how the coalition should engage with advocates across the state and with state-level issues? For instance, do you envision the NOLA MCH Coalition branching out in terms of scope? Do you think the coalition should be involved in helping to form MCH coalitions in other cities?

***5. Processes and procedures***

13. What is your impression of the processes the coalition uses to do its work? (From both an internal, and external perspective, if applicable)?

14. Are there any weaknesses or areas for improvement in how the coalition operates?

a. How can the coalition be more efficient in achieving its mission? What are some of the most creative and effective ways in which the coalition should operate within the New Orleans maternal and child health (or broader) landscape?

b. Do organizations of different sizes, budgets, types, and relationships to Tulane feel equally able to participate and help shape the direction of the coalition?

c. How does the affiliation with Tulane affect how people’s participation?

d. Are you aware of any other factors that might be constricting what agency representatives and members say or do in coalition meetings and communications (grant/funding relationships, race or social group, etc.)?

e. What is your impression of whether participants feel that their voices are heard? If not, why?

15. What steps or actions should the coalition prioritize right now? In the future?

***6. Other***

16. Is there anything I haven’t asked you about that you think we should be thinking about with respect to the work/role/future of the Coalition, and more broadly about building collective power to advance maternal and child health equity in the City of New Orleans?

17. Are there other key informants you think we should interview?
